# Mitotic arrest affects clustering of tumor cells

**DOI:** 10.1186/s13008-021-00070-z

**Published:** 2021-01-29

**Authors:** Julia Bonnet, Lise Rigal, Odile Mondesert, Renaud Morin, Gaëlle Corsaut, Mathieu Vigneau, Bernard Ducommun, Valérie Lobjois

**Affiliations:** 1 Université de Toulouse, ITAV, CNRS, ITAV-USR3505, 1 Place Pierre Potier, Toulouse Cedex 1, 31106 France; 2 IMACTIV-3D SAS, Toulouse, France; 3 CHU de Toulouse, Toulouse, France

**Keywords:** Cancer cell clustering, Mitosis, Microtubule-targeting agents, Anchorage-independent aggregation, Quantitative live imaging

## Abstract

**Background:**

Cancer cell aggregation is a key process involved in the formation of tumor cell clusters. It has recently been shown that clusters of circulating tumor cells (CTCs) have an increased metastatic potential compared to isolated circulating tumor cells. Several widely used chemotherapeutic agents that target the cytoskeleton microtubules and cause cell cycle arrest at mitosis have been reported to modulate CTC number or the size of CTC clusters.

**Results:**

In this study, we investigated in vitro the impact of mitotic arrest on the ability of breast tumor cells to form clusters. By using live imaging and quantitative image analysis, we found that MCF-7 cancer cell aggregation is compromised upon incubation with paclitaxel or vinorelbine, two chemotherapeutic drugs that target microtubules. In line with these results, we observed that MCF-7 breast cancer cells experimentally synchronized and blocked in metaphase aggregated poorly and formed loose clusters. To monitor clustering at the single-cell scale, we next developed and validated an in vitro assay based on live video-microscopy and custom-designed micro-devices. The study of cluster formation from MCF-7 cells that express the fluorescent marker LifeAct-mCherry using this new assay allowed showing that substrate anchorage-independent clustering of MCF-7 cells was associated with the formation of actin-dependent highly dynamic cell protrusions. Metaphase-synchronized and blocked cells did not display such protrusions, and formed very loose clusters that failed to compact.

**Conclusions:**

Altogether, our results suggest that mitotic arrest induced by microtubule-targeting anticancer drugs prevents cancer cell clustering and therefore, could reduce the metastatic potential of circulating tumor cells.

## Background

Metastatic dissemination of epithelial tumor cells that invade, circulate, and form a tumor at distant sites [1, 2] is a major challenge for cancer therapy. Circulating tumor cells (CTCs) are detected in patients’ blood samples, and CTC clusters have been associated with higher metastatic potential [3, 4]. Indeed, formation of tumor cell clusters prevents anoikis in the absence of anchorage and prolong their survival [5, 6]. Moreover, CTC clusters display higher metastatic potential than isolated CTCs and are associated with adverse outcomes [3, 7]. Their role in tumor dissemination suggests that they should be considered in anti-metastasis strategies [8–10]. Therefore, the clinical implementation of sensitive and reliable technologies to detect and quantify CTCs and CTC clusters is currently the subject of major interest (see for instance [11–13]). However, only few regulators of tumor cell clustering have been identified, and the underlying mechanisms remain unclear. For instance, plakoglobin, a cell junction component, is differentially expressed in breast cancer, its knockdown in mouse abrogates CTC cluster formation, and is a significant prognostic predictor [3, 14]. Similarly, it has been shown that breast cancer metastases arise from keratin 14-expressing tumor cell clusters [1]. We recently reported, using time-lapse microscopy-based clustering that E-cadherins and also desmoglein and desmocolin, two desmosomal proteins, are involved in cancer cell aggregation [15]. Using the same approach, we also demonstrated the involvement of gap junction intercellular communication in regulating the earliest step of tumor cell clustering [16].

Cell proliferation is tightly associated with the successful completion of the cell cycle that culminates with mitosis. Anti-mitotic drugs that impair or inhibit mitosis ultimately result in cell death, and effectively target and kill tumor cells [17]. Spindle poisons, such as vinca alkaloids, paclitaxel and related taxanes, target microtubule dynamics, resulting in mitotic arrest through the activation of the spindle assembly checkpoint [18]. These compounds are highly effective anti-cancer drugs in vitro and in clinical settings, and are currently used to treat many tumor types, including breast and ovarian metastatic cancers [19–21]. Interestingly, very recently, a screen of a FDA-approved compounds library identified tubulin polymerizing inhibitors for their ability to decrease the size of human breast CTC clusters [9]. However, a pro-metastatic effect of these compounds has also been reported in mouse mammary tumor models associated with an increase of circulating tumor cells [22, 23]. Indeed, the number of detected isolated circulating tumor cells increases massively after paclitaxel therapy indicating that tumor cells are mobilized from the primary tumor in response to chemotherapy.

Considering these data, we wondered whether tubulin polymerizing inhibitors, through their anti-mitotic effect, could impact the ability of tumor cells to aggregate and form clusters.

We first observed that paclitaxel and vinorelbine affect in vitro formation of MCF-7 clusters. We then used population and single-cell scale in vitro assays with MCF-7 cells experimentally blocked at metaphase and found that mitotic MCF-7 breast cancer cells form clusters that are poorly aggregated and unstructured. This impaired clustering was associated with rounding of mitotic cells and lack of actin-based membrane dynamics. We therefore propose that anticancer drug-induced cell cycle blockade at mitosis might modulate the metastatic potential of circulating tumor cells by reducing their clustering.

## Results

### MCF-7 cells incubated with paclitaxel or vinorelbine form clusters less efficiently

**Fig. 1:**
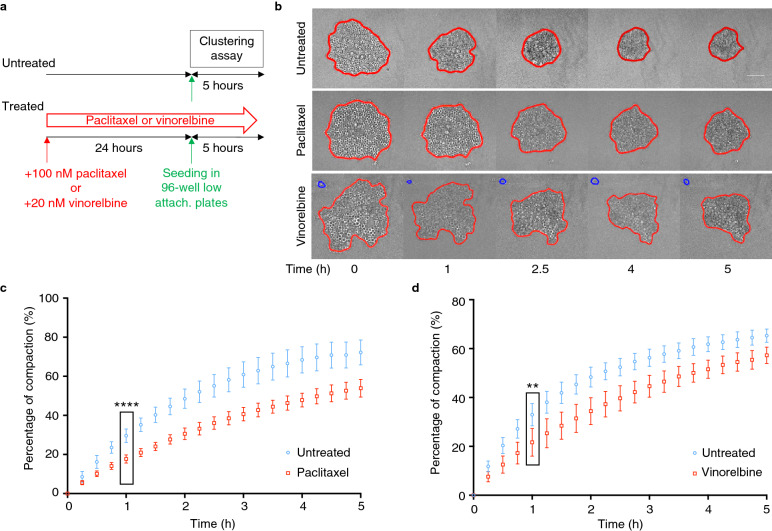
Aggregation is compromised in MCF-7 cells incubated with paclitaxel or vinorelbine. **a** Schematic representation of the experimental set-up. MCF-7 cells incubated or not (untreated) with paclitaxel (100 nM) or vinorelbine (20 nM) for 24 h were seeded in 96-well low-attachment (low-attach.) plates and monitored by video-microscopy for 5 h (clustering assay) (adapted from [15]). **b** Representative transmitted light microscopy images of cell aggregation at the indicated time points. Segmentation (red line) was performed using a dedicated MATLAB software. Blue lines correspond to isolated cells. **c**, **d** An automated image processing procedure was used to measure the aggregate area during the assay in the presence of paclitaxel (**c**) or vinorelbine (**d**) and the percentage of compaction was calculated from the normalized area variation relative to the initial time point. Data correspond to the mean ± SD of 48 aggregates/condition from 3 independent experiments. **P < 0.01; ****P < 0.0001 (Mann–Whitney non-parametric test)

Paclitaxel and vinorelbine are two microtubule-targeting anticancer drugs that induce cell cycle block at mitosis and inhibition of cell proliferation [20]. To assess their effect on MCF-7 cell ability to compact and form clusters, cells incubated with paclitaxel or vinorelbine for 24 h were subjected to a previously described aggregation assay in which the progressive aggregation and compaction of 500 cells seeded in non-adherent 96-well plates are monitored by video-microscopy for 5 h [15, 16] (Fig. 1a). Cells incubated with 100 nM paclitaxel or 20 nM vinorelbine for 24 h accumulated in mitosis with very limited cell death, as confirmed by flow cytometry analysis (data not shown). Figure 1b shows representative images of the clustering and compaction kinetics of control and treated cells. To quantify compaction over time, the cluster area was determined at each time point by automated image segmentation (red line) with a custom-designed MATLAB routine. Using this quantification, we found that clustering of MCF-7 cells incubated with paclitaxel or vinorelbine was altered, and that at 5 h, compaction was reduced by about 25% compared with untreated cells (Fig. 1c, d).

### Cluster formation is reduced in metaphase-synchronized MCF-7 breast cancer cells

**Fig. 2:**
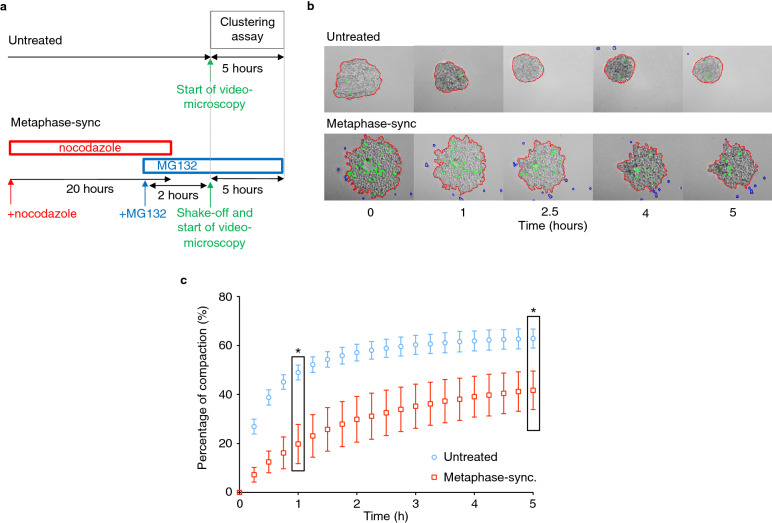
Anchorage-independent aggregation is inhibited in metaphase-blocked MCF-7 breast cancer cells. **a** Schematic representation of the synchronization procedure. Cells were incubated with nocodazole for 20 h, and MG132 was added to the medium for the last 30 min. Then, the culture medium was replaced by medium containing only MG132 for 1.5 h before mitotic shake-off and initiation of the aggregation assay of cells arrested in mitosis to monitor their clustering. **b** Control (untreated) and metaphase-synchronized MCF-7 cells were seeded in 96-well low-attachment plates and monitored by video-microscopy for 5 h. Representative transmitted light microscopy images of cell aggregation at the indicated time points. Segmentation (red line) was performed using a dedicated MATLAB software. Green lines correspond to the excluded holes, and blue to isolated cells. **c** Using the automated image processing data, the aggregate area was measured over time. The graph corresponds to the percentage of compaction calculated from the normalized area variation relative to the initial time point. Data correspond to the mean ± SD of 48 aggregates for each condition from 3 independent experiments. *P < 0.001 (Mann–Whitney non-parametric test)

Considering the observed reduction of cancer cell compaction induced by paclitaxel and vinorelbine, we examined the effect of mitotic arrest on the cancer cell ability to form clusters. We first monitored cluster formation in MCF-7 breast cancer cells synchronized and blocked in metaphase. To this aim, we incubated cells with nocodazole for 20 h followed by culture with the proteasome inhibitor MG132 for 2 h to synchronize cells at the metaphase-anaphase checkpoint and by a final shake-off step to retain only mitotic cells [24] (see “Methods” and Fig. 2a for details). Flow cytometry analysis of the cell cycle distribution performed after the shake-off indicated that about 85% of cells were arrested in metaphase. This was also confirmed by immunofluorescence analysis with an anti-α-tubulin antibody that showed metaphase arrested cells with an organized bipolar mitotic spindle (Additional file 1: Fig. S1a, b).

Then, we used the same aggregation assay described previously. Figure 2b shows representative micrographs of the aggregation kinetics of control (no synchronization) and metaphase-synchronized MCF-7 cells. The quantification (Fig. 2c) of these data indicated that MCF-7 cell clustering was much slower and cell compaction to form cluster was reduced by approximately 50% in metaphase-blocked MCF-7 tumor cells compared with control cells. In the same conditions, MG132 alone slightly affected the aggregation dynamics of MCF-7 cells, but to a much lower extent than synchronization in metaphase (Additional file 2: Fig. S2). We also showed that microtubule cytoskeleton disruption has no effect on MCF-7 cell aggregation dynamics (Additional file 3: Fig. S3) and that the aggregation ability of cells accumulated in mitosis independently of microtubule cytoskeleton disruption is also altered (Additional file 4: Fig. S4), suggesting that the accumulation in mitosis is associated with lower ability of MCF-7 tumor cells to aggregate and form clusters independently of microtubule depolymerization.

### Cohesion of aggregates formed by metaphase-blocked MCF-7 cells is strongly reduced

**Fig. 3:**
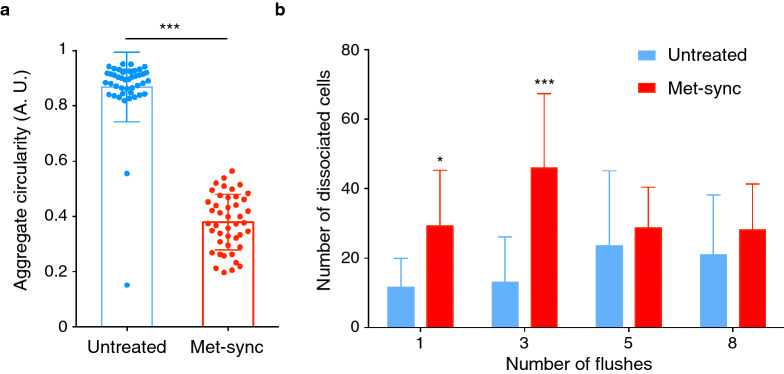
Metaphase-blocked MCF-7 breast cancer cells form less cohesive clusters. **a** Aggregate circularity was determined in control (untreated) and metaphase-synchronized/blocked MCF-7 cells after 5 h. ***P < 0.001 (Mann–Whitney non-parametric test). **b** Cluster cohesion analysis using a flush-assay. After 5 h, clusters were mechanically dissociated by repeatedly flushing with a micropipette. The number of dissociated cells was determined. Data are the mean ± SD of 12 wells for each condition from 4 independent experiments. *P < 0.05; ***P < 0.001 (two-way ANOVA)

As observed in Fig. 2, aggregates formed by metaphase-blocked cells after 5 h were less round than those formed by control asynchronous cells. This was confirmed by the finding that the aggregate circularity (Fig. 3a) was significantly lower in metaphase-blocked than in control MCF-7 tumor cells (mean value: 0.38 ± 0.1 versus 0.87 ± 0.02). This suggests that metaphase-blocked cells aggregate more slowly than control cells and form looser and less cohesive clusters. Therefore, we assessed the aggregate cohesion by using a dissociation assay in which the cell aggregate cohesion is mechanically challenged by a specific number of sequential aspirations and flushes performed using a micropipette. We used the number of released cells as an indicator of the aggregate cohesion (see “Methods” for details). The number of cells released from control cell aggregates was small (Fig. 3b), indicating that strong intercellular interactions were already established after 5 h. Conversely, metaphase-blocked cell aggregates were rapidly and fully dissociated, indicating that the less efficient aggregation of metaphase-synchronized cells was associated with the formation of less cohesive clusters by poorly adherent cells.

### An original assay to investigate clustering at the single-cell scale

**Fig. 4:**
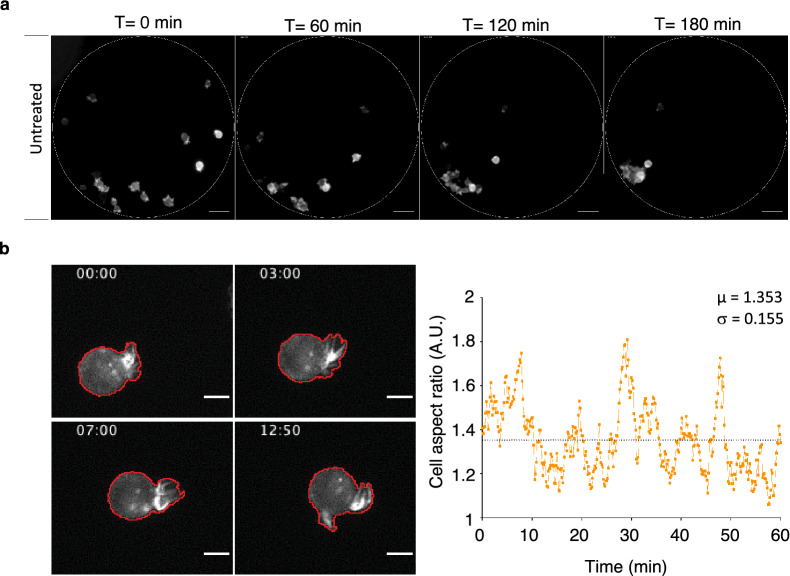
Monitoring MCF-7 cancer cell clustering at the single-cell scale. MCF-7 cells that express the LifeAct-mCherry fluorescent reporter were seeded in PDMS micro-wells placed in culture dishes (Additional file 2: Fig. S2) and their clustering was monitored by video-microscopy. **a** Representative fluorescence images of control (untreated) MCF-7 cells at different time-points during clustering. The dotted line shows the micro-well edge. Scale bar: 50 µm. **b** Representative micrographs of a MCF-7 cell that express the LifeAct-mCherry fluorescent reporter during aggregation (left panels). Membrane protrusions were automatedly detected (red line). Time is indicated in min. Scale bar: 10 µm. (Right panel) Plot of the aspect ratio of a single cell with mean value (µ) and standard deviation (σ) over 1 h

The previous aggregation assay does not allow assessing cell–cell interaction at the single-cell scale due to the high cell density. Therefore, we developed a new video-microscopy assay to analyze the dynamics of anchorage-independent clustering in single MCF-7 cells. To this aim, we designed and produced dedicated PDMS micro-wells (see “Methods” and Additional file 5: Fig. S5) that were treated with pluronic acid, a non-ionic surfactant to prevent cell anchorage to the substrate. Then, we seeded MCF-7 cells that express the LifeAct-mCherry fluorescent reporter in these micro-wells at a concentration that allowed the sedimentation of about 15-20 cells/microwell (Fig. 4a). Time-lapse acquisition (see Additional file 6: Movie S1 and representative fluorescence images in Fig. 4a) showed that within 3 h, untreated cells formed several small clusters that progressively gathered together, resulting in a main single compact cluster. In these experimental conditions, we observed that during aggregation, control MCF-7 cells formed large and highly dynamic protrusions that could be visualized by video-microscopy thanks to the LifeAct-mCherry fluorescent reporter (Fig. 4b, left panels and Additional file 7: Movie S2). The dynamics of these large protrusions could be captured by determining the aspect ratio of single cells that were individually analyzed during the first hour of the clustering experiment (Fig. 4b, right panel). From these data, we determined the mean value (µ) of the aspect ratio (AR) and the standard deviation (σ) of the AR values of each cell during the first hour as an indicator of the cell shape changes over time.

### Protrusions associated with anchorage-independent self-aggregation are dependent on the actin cytoskeleton

**Fig. 5:**
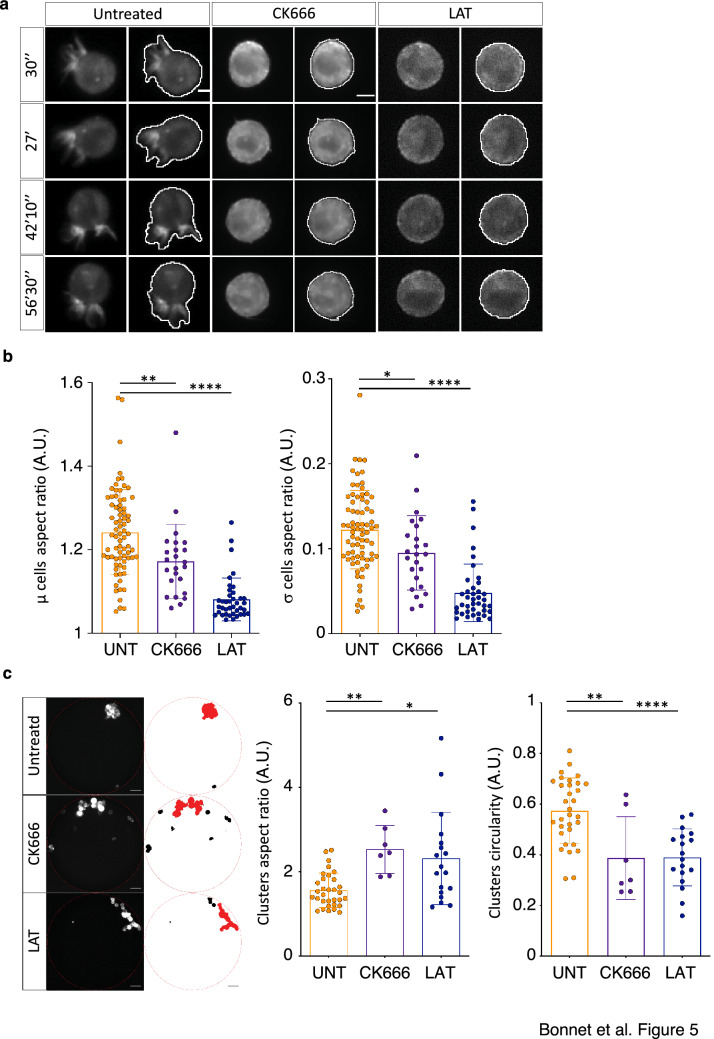
Membrane protrusions require a functional actin cytoskeleton. **a** Representative micrographs of MCF-7 cells that express the LifeAct-mCherry fluorescent reporter and incubated or not (untreated) with CK666 or latrunculin A (LAT) at different time-points during aggregation. Cell contours and membrane protrusions were automatedly detected (white line). Scale bar: 10 µm. **b** Determination of the average (µ) and standard deviation (σ) aspect ratio of single cells in control (untreated; UNT), and in cells incubated with CK666 or latrunculin A (LAT). *P < 0.05; **P < 0.01; ****P < 0.0001 (Mann–Whitney non-parametric test). Each dot corresponds to the values of one cell. **c** Graphs showing the aspect ratio and circularity of the larger clusters (see micrographs in left panels, larger clusters are in red) in control (UNT) and CK666 or latrunculin (LAT)-treated cells after 3 h of clustering. Each dot corresponds to the values of one well. For b and c, data are from 5 independent experiments, and bars correspond to the mean ± SD. *P < 0.05; **P < 0.01; ****P < 0.0001 (Mann–Whitney non-parametric test)

Considering the crucial role of actin filament polymerization in driving cell shape dynamics and motility, we incubated MCF-7 cells, during the clustering assay, with latrunculin A, which disrupts microfilament organization by binding to monomeric G-actin, and with CK666, an inhibitor of the F-actin nucleator complex Arp2/3 [25]. In asynchronous (control) cells, latrunculin A and CK666 abolished cell protrusion formation (Fig. 5a, and Additional file 8: Movie S3 and Additional file 9: Movie S4) and protrusion dynamics, as indicated by the significative decrease of the mean and standard deviation of the AR compared with untreated cells (Fig. 5b). Moreover, at the end of the clustering experiments (3 h), cell aggregation was impaired in asynchronous cells incubated with CK666 or latrunculin A, as shown by the significantly higher AR and lower circularity of the formed clusters (Fig. 5c). These results indicate that inhibition of actin-dependent cell protrusion formation and dynamics is associated with less efficient clustering.

### Metaphase-blocked MCF-7 cells do not develop large protrusions during anchorage-independent aggregation

**Fig. 6:**
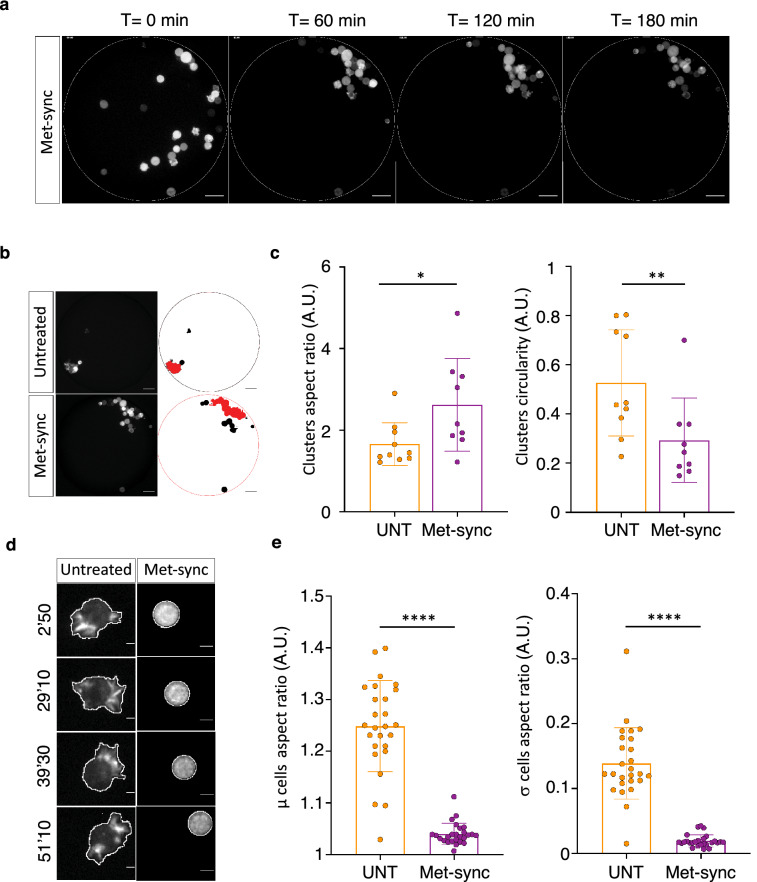
Metaphase-blocked MCF-7 cells do not form actin-dependent protrusions during anchorage-independent aggregation. **a** Representative fluorescence images of metaphase-synchronized/blocked MCF-7 cells at different time-points during clustering. The dotted line shows the micro-well edge. Scale bar: 50 µm. **b** Left: Images of a PDMS micro-well with untreated cells (top) and metaphase-synchronized/blocked MCF-7 cells (Met-sync, bottom) at the last time point of the time-lapse experiment (180 min). Right: Corresponding binary images at the end of the image segmentation process. Circles, edge of the micro-well; red, the largest cluster formed that was used for the aspect ratio and circularity analysis. **c** Graphs showing the aspect ratio (left) and circularity (right) analysis results for the larger clusters in control (untreated; UNT) and metaphase-synchronized (Met-sync) cells after 3 h of clustering. Each dot corresponds to the values in one micro-well from 5 independent experiments and bars correspond to the mean ± SD. *P < 0.05; **P < 0.01 (Mann–Whitney non-parametric test). **d** Representative micrographs of control (untreated) and metaphase-synchronized/blocked (Met-sync) MCF-7 cells that express the LifeAct-mCherry fluorescent reporter during aggregation (Left panels). The white line shows the region of interest (ROI) used for the aspect ratio determination. **e** Graphs showing the average (µ) and standard deviation (σ) aspect ratio of control (UNT) and in metaphase-synchronized/blocked cells (Met-sync) after 1 h of aggregation. Each dot corresponds to one cell and the bars correspond to the mean ± SD. Data are from 5 independent experiments with 5-6 cells analyzed per experiment. ****P < 0.0001 (Mann–Whitney non-parametric test)

In the microdevice-based assay, metaphase-synchronized cells (Fig. 2a) started to form a cluster, but they did not organize and efficiently compact (Fig. 6a). Quantitative analysis of the shape of the final cluster in each microwell (Fig. 6b, c) by determining its AR and circularity showed that control (asynchronized) cells formed a compact structure (mean AR of 1.6 and mean circularity of 0.5 for untreated cells) as well as cells incubated only with nocodazole or MG132 (Fig. 6c and Additional file 10: Fig. S6a, b). Conversely, metaphase-blocked cells formed unstructured clusters (mean AR of 2.5 and mean circularity of 0.3). In these conditions, we observed that during anchorage-independent aggregation metaphase-blocked cells did not form dynamic membrane protrusions (Fig. 6d). Comparison of the AR in control (asynchronous) and metaphase-arrested single cells (Fig. 6e) confirmed this observation. Incubation with nocodazole or MG-132 alone did not affect protrusion formation (Additional file 10: Fig. S6c, d).

These results clearly show that, in these experimental conditions, metaphase-blocked cells aggregate significantly less efficiently and form loose clusters of poorly associated cells. As shown with actin-targeting drugs, less efficient clustering is associated with absence of dynamic cell protrusion formation.

## Discussion

During the process of metastasis, cancer cells escape from the primary tumor and reach distant sites through the blood or lymphatic vessels. These cells are known as CTCs. Furthermore, clusters of circulating tumor cells have also been detected in the blood of patients with metastatic cancers. These clusters have an increased metastatic potential compared to single CTCs and their presence correlates with poor prognosis [3]. A recent study identified a reduction in size of CTC clusters after treatment with tubulin-binding drugs [9]. Several studies also revealed variations of CTC number after treatments with anticancer drugs that target the microtubule cytoskeleton [22]. Altogether, these data suggest that treatments with these anti-mitotic drugs might affect CTC cluster formation and stability.

In this study, we investigated in vitro how anti-mitotic agents could influence the clustering and aggregation of mitotic MCF-7 cancer cells in anchorage-independent conditions using dedicated microdevices and time-lapse video microscopy.

First, by video-microscopy analysis of the aggregation and compaction of a large cell population over time, we showed that anchorage-independent aggregation is inhibited by incubation with paclitaxel and vinorelbine and also in metaphase-blocked mitotic tumor cells. Then, using a mechanical assay, we found that the cohesion of aggregates formed by mitotic cells is dramatically reduced. Finally, we developed a new assay in which an array of PDMS micro-wells allows monitoring and studying the clustering of single cancer cells in the absence of anchorage. In these conditions, again, we found that MCF-7 cell aggregation is less efficient and does not result in compact aggregates, but rather in loose clusters. These results strongly suggest that in MCF-7 cells blocked in mitosis, the capacity to aggregate and form compact clusters is reduced.

We showed that during anchorage-dependent clustering, MCF-7 cells form large and dynamic actin-dependent protrusions, and that alteration of their formation by using actin-targeting drugs alters cluster formation in PDMS micro-wells. This result is consistent with our previous findings showing that latrunculin A affects cell aggregation in ultra-low attachment plates [16]. As our experiments were performed using ultra-low attachment plates on which cells could not adhere, the cell shape modifications during cluster formation could not be attributed to adherence to the substratum and migration. MCF-7 cells synchronized and blocked in metaphase do not form protrusions. Rounding at mitosis is associated with elevated intracellular pressure and recruitment of myosin to the cell cortex that leads to an increase in cortical tension, which is maximal at metaphase [26, 27]. Moreover, we previously demonstrated that cytoskeleton tension due to myosin IIa acts as an inhibitor of cell aggregation [15]. Therefore, we hypothesized that less efficient clustering of metaphase-synchronized cells could be due to a decrease in formation and/or dynamics of protrusions, as a consequence of cortical tension increase. Accordingly, the absence of protrusion in MCF-7 cells synchronized and blocked in metaphase suggests that acto-myosin dependent cortical tension could also be an important parameter in tumor cell cluster formation.

Dynamic microtubule-based protrusions, named micro-tentacles, have been described also in breast carcinoma cell lines [28]. The formation of these extensions is counteracted by the actin-cytoskeleton and these microtubule-dependent plasma membrane extensions have been associated with tumor cell retention in the lung vasculature. Here, we found that nocodazole does not affect actin-dependent protrusion formation and aggregation dynamics in MCF-7 cells, showing that different mechanisms involving the plasma membrane could contribute to CTC survival and metastatic potential during their journey from the primary tumor to the vasculature, clustering, and formation of metastases.

The prognostic value and clinical relevance of CTC enumeration and its association with progression-free survival (PFS) in advanced-stage breast cancer is now well established, and the presence of CTC clusters has been associated with shorter PFS [29]. CTC clusters provide additional prognostic value compared with CTC count alone [30–32]. The effects of microtubule-targeted agents on CTC biology is not fully understood. As stated above, several publications showed that CTC number increases massively upon therapy, suggesting that they can be rapidly mobilized from the primary tumor after treatment with paclitaxel [22]. As discussed by others, this should be carefully examined to ensure that antimitotic therapies do not increase the metastatic potential [28]. It has been reported that taxane treatment enhances tumor cell reattachment [33]. However, to date no data has been published on the increase of isolated CTCs and clusters upon treatment with paclitaxel and related compounds [22]. On the basis of our findings, we might hypothesize that paclitaxel therapy while leading to CTC rapid increase could also decrease their capacity to form clusters, thus resulting in an overall reduction of the metastatic risk.

## Conclusions

In summary, metaphase-blocked cells initiate spontaneous aggregation in anchorage-independent in vitro experimental conditions, but fail to form compact clusters and associated dynamic actin-dependent protrusions. In line with these results, incubation with the anticancer tubulin-targeting drugs paclitaxel and vinorelbine alters MCF-7 tumor cell cluster formation. These results provide insights into the possible effect of anti-mitotic chemotherapeutic agents on CTC metastatic potential.

## Methods

### Cell culture

MCF-7 cells (ATCC HTB-22) were cultured in RPMI (Gibco, Life Technologies) supplemented with 1 µmol/L insulin (Sigma Aldrich), 10% fetal calf serum (FCS) (Gibco, Life Technologies) and 1% penicillin/streptomycin (100 U/ml, Gibco, Life Technologies) in a humidified atmosphere of 5% C0_2_ at 37 °C. For time-lapse microscopy imaging, culture medium was replaced by OPTIMEM + Glutamax (Gibco by Life Technologies) supplemented with 1 µmol/L insulin, 10 nmol/L ß-estradiol, 20 ng/ml epidermal growth factor (Invitrogen), B-27 Supplement (1X, Invitrogen), 1% penicillin/streptomycin (100 U/ml, Gibco, Life technologies).

To study the impact of paclitaxel and vinorelbine, cells were incubated in culture medium containing 100 nM paclitaxel or 20 nM vinorelbine for 24 h, then trypsinized and seeded for aggregation assay in culture medium containing the same concentrations of paclitaxel and vinorelbine. For synchronization in mitosis, cells were incubated with 200 ng/ml nocodazole for 20 h to accumulate in an abnormal pro-metaphase state. Cells were then incubated in culture medium containing 200 ng/ml nocodazole and 25 µM MG132 for 30 min, and then in medium containing only 25 µM MG132 for 1.5 h, according to the protocol described by Cazales et al. [24]. Addition of MG132 blocked cells in metaphase by inhibiting sister chromatid separation. Mitotic shake-off [34] was used to select only mitotic cells that were used for the clustering and aggregation assays. For control experiments, MG132 (25 µM) or nocodazole (200 ng/ml) were added to the culture medium just before seeding for aggregation assays. For actin cytoskeleton inhibition, latrunculin A (200 nM, Sigma) and CK666 (150 µM, Sigma) were added to the culture medium just before seeding for aggregation assays.

### Immunofluorescent staining

Cells were grown on coverslips coated with poly-l-lysine. Cells were washed in PBS, fixed for 10 min in formalin (Sigma) then washed and permeabilized in PSB/0.25% Triton X-100 for 5 min at room temperature, and incubated in PBS/1%BSA 30 min at room temperature. Coverslips were then incubated at 37 °C with anti-tubulin antibodies (1:2000, Sigma #T5168) in PBS/0.1%BSA for 1 h. After washes in PBS, goat anti-mouse Alexa 488 antobodies (1/800, Molecular probes # A-11001) were applied at room temperature for 1 h. DNA was stained with DAPI at 1 µg/ml at room temperature for 10 min.

### LifeAct-mCherry-expressing MCF-7 cell line

The 17-amino acid LifeAct coding sequence fused to GFP2 was excised from the pLifeAct-TagGFP2 vector (Ibidi; catalog number#60101) and cloned in the pTRIP lentiviral shuttle vector in frame with the cDNA encoding the mCherry fluorescent protein. The resulting plasmid (pTRIP LifeAct mCherry) was used to produce lentiviral particles in 293FT embryonic kidney cells (Life Technologies) after calcium chloride transfection with the pGag/pol and pVSV-G plasmids (provided by the Vectorology platform, INSERM U1037) [35]. At 7 h post-transfection, DMEM + Glutamax (Gibco by Life Technologies) complemented with 10% FCS was washed off and replaced with serum-free OPTIMEM + Glutamax (Gibco by Life Technologies). Lentiviral particles were harvested 48 h later and the viral titer was quantified by flow cytometry (BD Accuri C6) in HT1080 cells (ATCC) transduced with serial dilutions of lentiviruses. MCF-7 cells (ATCC HTB-22) were then transduced in the presence of 4 μg/ml protamine sulfate in OPTIMEM + Glutamax. The medium was replaced 7 h later by RPMI (Gibco by Life Technologies) supplemented with 10% FCS and 1 µM insulin (Sigma-Aldrich, Ref. I0516). The generated stable LifeAct-mCherry-expressing MCF-7 cell line underwent two rounds of cell sorting (Cytometry and Cell Sorting platform, INSERM UMR 1048) followed by single-cell clonal isolation in 96-well plates.

### Flow cytometry analyses

Trypsinized cells were collected and fixed in 4% formalin solution (Sigma-Aldrich) for 10 min, then washed and permeabilized in PBS/1% BSA containing 0.25% Triton X-100 on ice for 5 min. Mitotic cells were detected with the 3.12.i.22 antibody [36] diluted (1:10000) in PBS/0.1% BSA. After a wash in PBS, cells were incubated with a goat anti-mouse Alexa Fluor 488 antibody (Molecular Probes) at room temperature for 1 h. After DNA staining with 10 μg/mL propidium iodide (Sigma Aldrich) at room temperature for 30 min, cells were analyzed with an Accuri™ C6 Flow Cytometer (BD Science) and the Accuri software.

### Aggregation assay

This assay was performed essentially as previously described [15, 16]. Cells (500 cells/well) were seeded in low-attachment round-bottomed 96-well plates (Costar^®^), except in the 36 peripheral wells to avoid edge effects. Plates were centrifuged at 400 g for 4 min, and then cell aggregation in each well was followed by time-lapse video-microscopy. Images were acquired with an inverted widefield Zeiss Axio Observer microscope fitted with a 0.3 N.A. 10X objective and a CoolSNAP CDD camera (Roper scientific) in bright-field for at least 5 h (1 acquisition/15 min). At each time point and position, 20-µm spaced z-stacks over 160 µm depth (8 stacks) in brightfield were acquired. A custom-made MATLAB procedure was used to monitor and measure cell cluster formation over time. The main steps of the workflow were: (1) image processing at each time point and for each cluster by focus stacking to merge images of multiple focal planes into one in-focus image; (2) binarization and edge detection with a Sobel filter to define the boundaries of each cluster and of holes inside the cluster (to exclude them); (3) saving the projection, segmentation and image overlay; and (4) calculation of the typical parameters (perimeter, area, normalized area: Area T0/Area T(x)).

### Evaluation of aggregate cohesion—Flush assay

Aggregates formed in each well were mechanically dissociated directly in the wells by making 1, 3, 5, or 8 flushes. In each flush, 50 µl of cells and medium were gently aspirated with a multi-channel micropipette and vigorously flushed back. Cells were then allowed to sediment for 10 min before quantifying the number of dissociated individual cells in 10µL. Quantification was done in triplicate for each experimental condition.

### Single-cell clustering assay in dedicated PDMS micro-wells

The PDMS pre-polymer was mixed with the polymerization agent Sylgard 184 (10:1 ratio), degassed in a vacuum chamber, and poured in a silicon wafer (RENATER facility of LAAS, CNRS, France). After a second degassing, PDMS was cured at 60 °C overnight. Arrays of nine PDMS micro-wells (see Additional file 2: Fig. S2) were cut, peeled off, and glued in each compartment of CELLview^TM^ cell culture dishes (Greiner Bio-one). Micro-wells were incubated with 20 mg/ml Pluronic-F127 (Sigma) overnight to prevent cell adhesion, and then rinsed twice before use.

LifeAct-mCherry-expressing MCF-7 cells were distributed in the compartments at a density that allowed the sedimentation of approximately 20 cells per micro-well. Cluster formation was followed by time-lapse video-microscopy using an inverted widefield Zeiss Axio Observer microscope fitted with a 0.3 N.A. 10X objective. Images were acquired for 3 h (one acquisition every 10 s) and processed with lmageJ software packages [37]. Before automated analysis, images were manually corrected. Specifically, parts of other cells, staining background and debris were removed using the clearing function of ImageJ. Then, the lmageJ macro was used for image segmentation and calculation of the shape descriptors (circularity and aspect ratio).

### Statistical analysis

Data were analyzed with GraphPad Prism version 6.00 (GraphPad soft- ware, La Jolla California USA, www.graphpad.com).

## Supplementary information

**Additional file 1: Figure S1.** Synchronization procedure. **a** Flow cytometry analysis of control (untreated) and mitosis-arrested cells (nocodazole/MG132). Mitotic cells were detected with the mitotic-specific monoclonal 3-12-I-22 antibody. **b** Representative fluorescence microscopy images (DAPI and α-tubulin) of cells blocked in mitosis (nocodazole/MG132) and used for the clustering assays. Scale bar: 10 µm.

**Additional file 2: Figure S2.** Impact of incubation with MG132 alone on MCF-7 cells clustering. **a** Control (untreated) and MG132-treated MCF-7 cells were seeded in 96-well low-attachment plates and monitored by video-microscopy for 5 h. Representative transmitted light microscopy images of cell aggregation at the indicated time points. Segmentation (red line) was performed using a dedicated MATLAB software. Green lines correspond to the excluded holes, and blue to isolated cells. **b** Using the automated image processing data, the aggregate area was measured over time. The graph corresponds to the percentage of compaction calculated from the normalized area variation relative to the time 0. Data correspond to the mean ± SD of 48 aggregates for each condition from 3 independent experiments. **a** and **b** The data of the Fig. 2 obtained with metaphase-synchronized cells are shown for comparison.

**Additional file 3: Figure S3.** MCF-7 cell clustering occurs independently of microtubules cytoskeleton disruption. **a** Immunostaining of -tubulin on control untreated MCF-7 cells (Control) and cells incubated with 10 µM nocodazole for 2 h. For both conditions, each row corresponds to two different fields of view. Inserts show the higher magnification of the region outlined with dotted lines in the corresponding image. *mitotic cells in control and nocodazole conditions. Scale bar: 10 µm. **b** Schematic representation of the experiment. To test the impact of microtubule depolymerization on aggregation dynamics, MCF-7 cells were pre-treated with 10 µM nocodazole for 1 h, then they were seeded in 96-well low-attachment plates in presence of 10 µM nocodazole, and monitored by video-microscopy for 5 h (clustering assay). **c** Percentage of compaction calculated from the normalized area at each time points (see “Methods” section) for control (untreated) cells and cells incubated with nocodazole, as described in b. For each time point, data correspond to the mean ± SD of 32 aggregates/condition from 3 independent experiments.

**Additional file 4: Figure S4.** Aggregation ability of cells accumulated in mitosis independently of microtubule cytoskeleton disruption is also altered. **a** Flow cytometry analysis of control (untreated) cells, cells incubated with 6 µM RO3306 (RO3306) for 20 h, or cells at the indicated time points after RO3306 removal from the culture medium. The upper panels show the histograms of the propidium iodide fluorescence intensity (DNA content) and the lower panels show the dot plots of DNA content versus intensity for the detection of the mitotic marker 3.12.I.22 (see “Methods” section). The percentage of mitotic cells (shown in green) is indicated for each condition. **b** Schematic representation of the experimental design for the aggregation assay. **c** The percentage of compaction was calculated at each time point of the clustering assay in control cells and in cells collected by shake-off at 2 h after RO3306 removal. Data correspond to the mean ± SD of 32 aggregates in control and 35 aggregates in treated cells from 3 independent experiments.

**Additonal file 5: Figure S5.** Microdevice to study clustering at the single-cell scale**. a** Mask used for the fabrication of the silicon wafer. **b** One array of 9 PDMS micro-wells (outer diameter: 650 µm, inner diameter: 450 µm, and height: 200 µm) that are (**c**) glued on the bottom of the compartments of CELLview^TM^ cell culture dishes for monitoring by time-lapse video-microscopy.

**Additional file 6: Movie S1.** Cell clustering in the microdevice. Time-lapse image acquisition of MCF-7 cells that express the LifeAct-mCherry fluorescent reporter during clustering. Transmitted and mCherry fluorescence images are merged. Movie duration: 3 h. Scale bar: 50 µm.

**Additional file 7: Movie S2.** Kinetics of one MCF-7 cell during clustering in a PDMS micro-well. Fluorescence images from time-lapse acquisition of one control MCF-7 cell that expresses the LifeAct-mCherry fusion protein. On the right panel, the white line corresponds to the ROI used for morphometric parameter determination. Scale bar: 10 µm.

**Additional file 8: Movie S3.** Kinetics of one MCF-7 cell incubated with CK666 during clustering in a PDMS micro-well. Fluorescence images from time-lapse monitoring of one CK666-treated MCF-7 cell that expresses the LifeAct-mCherry fusion protein. On the right panel, the white line corresponds to the ROI used for morphometric parameter determination. Scale bar: 10 µm.

**Additional file 9: Movie S4.** Kinetics of one latrunculin A-treated MCF-7 cell during clustering in a PDMS micro-well. Fluorescence images from time-lapse monitoring of one latrunculin A-treated MCF-7 cell that expresses the LifeAct-mCherry fusion protein. On the right panel, the white line corresponds to the ROI used for morphometric parameter determination. Scale bar: 10 µm.

**Additional file 10: Figure S6.** Characterization of clusters in control and experimental conditions. **a, b** Graphs showing the aspect ratio (**a**) and circularity (**b**) analysis results for the larger clusters formed in micro-wells in MCF-7 cells incubated or not (untreated, UNT) with nocodazole and MG132 (i.e., metaphase-synchronized/blocked, Met-sync), or with MG132 (MG) or nocodazole (Noco) alone after 3 h of clustering. Each dot corresponds to the values in one micro-well from 5 independent experiments and bars correspond to the mean ± SD. **c**, **d** Determination of the average (µ) (**c**) and standard deviation (σ) (**d**) aspect ratio in single MCF-7 cells incubated or not (untreated, UNT) with nocodazole and MG132 (i.e., metaphase-synchronized/blocked, Met-sync), or with MG132 (MG) or nocodazole (Noco) alone after 1 h of aggregation. Each dot corresponds to one cell and the bars correspond to the mean ± SD. Data are from 5 independent experiments with 5-6 cells analyzed per experiment. *P < 0.05; **P, < 0.01; ****P < 0.0001 (Mann–Whitney non-parametric test).

## Data Availability

All data generated or analyzed during this study are included in this published article and its supplementary information files.

## Abbreviations

CTC: Circulating tumor cell
PDMS: Polydimethyl siloxane
