# Supplementary material for: Mitotic arrest affects clustering of tumor cells
Source: Cell Div. 2021 Jan 29;16:2. doi: 10.1186/s13008-021-00070-z (PMC7847029; doi:10.1186/s13008-021-00070-z)
Supplement: Supplementary file 1 — Additional file 1: Figure S1. Synchronization procedure. a Flow cytometry analysis of control (untreated) and mitosis-arrested cells (nocodazole/MG132). Mitotic cells were detected with the mitotic-specific monoclonal 3-12-I-22 antibody. b Representative fluorescence microscopy images (DAPI and α-tubulin) of cells blocked in mitosis (nocodazole/MG132) and used for the clustering assays. Scale bar: 10 µm. [file 13008_2021_70_MOESM1_ESM.pdf]

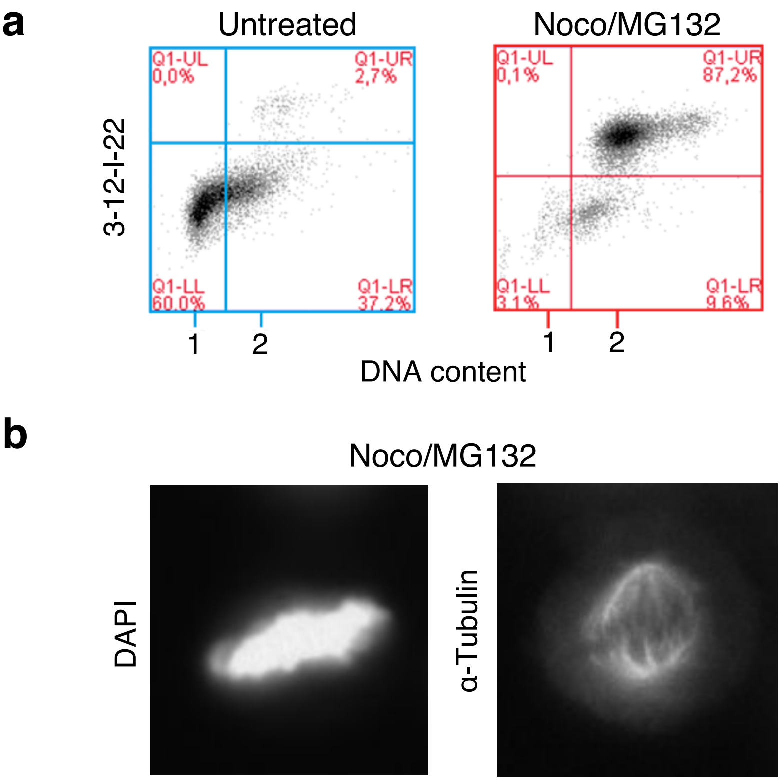

Supplementary Figure S1. Synchronization procedure

a Flow cytometry analysis of control (untreated) and mitosis-arrested cells (nocodazole/MG132). Mitotic cells were detected with the mitotic-specific monoclonal 3-12-I-22 antibody.

b Representative fluorescence microscopy images (DAPI and α-tubulin) of cells blocked in mitosis (nocodazole/MG132) and used for the clustering assays. Scale bar: 10µm.
