# Supplementary material for: Mitotic arrest affects clustering of tumor cells
Source: Cell Div. 2021 Jan 29;16:2. doi: 10.1186/s13008-021-00070-z (PMC7847029; doi:10.1186/s13008-021-00070-z)
Supplement: Supplementary file 2 — Additional file 2: Figure S2. Impact of incubation with MG132 alone on MCF-7 cells clustering. a Control (untreated) and MG132-treated MCF-7 cells were seeded in 96-well low-attachment plates and monitored by video-microscopy for 5 h. Representative transmitted light microscopy images of cell aggregation at the indicated time points. Segmentation (red line) was performed using a dedicated MATLAB software. Green lines correspond to the excluded holes, and blue to isolated cells. b Using the automated image processing data, the aggregate area was measured over time. The graph corresponds to the percentage of compaction calculated from the normalized area variation relative to the time 0. Data correspond to the mean ± SD of 48 aggregates for each condition from 3 independent experiments. a and b The data of the Fig. 2 obtained with metaphase-synchronized cells are shown for comparison. [file 13008_2021_70_MOESM2_ESM.pdf]

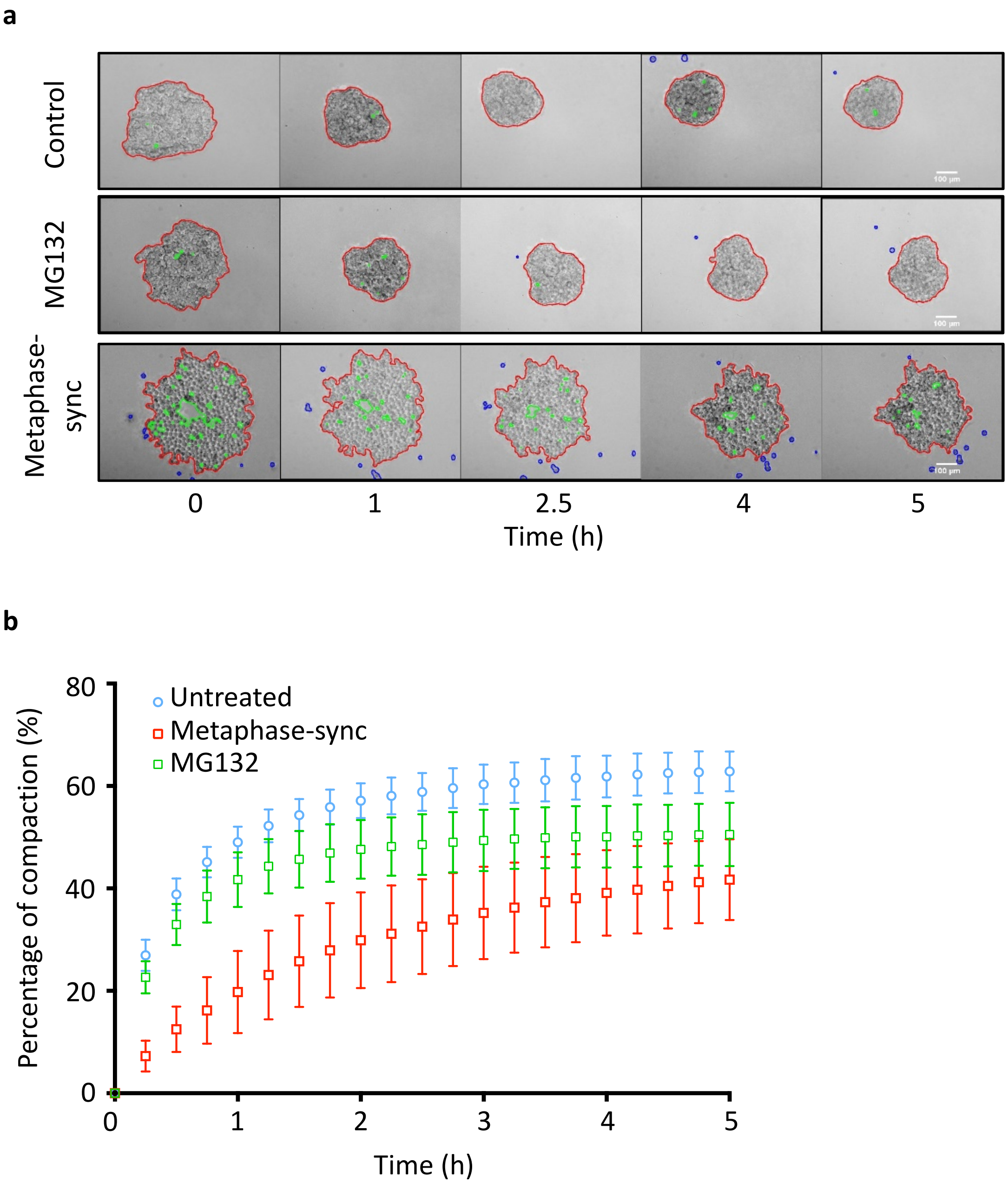

Supplementary Figure S2. Impact of incubation with MG-132 alone on MCF-7 cells clustering.

a Control (untreated) and MG132-treated MCF-7 cells were seeded in 96-well low-attachment plates and monitored by video-microscopy for 5 hours. Representative transmitted light microscopy images of cell aggregation at the indicated time points. Segmentation (red line) was performed using a dedicated MATLAB software. Green lines correspond to the excluded holes, and blue to isolated cells. **b** Using the automated image processing data, the aggregate area was measured over time. The graph corresponds to the percentage of compaction calculated from the normalized area variation relative to the time 0. Data correspond to the mean ± SD of 48 aggregates for each condition from 3 independent experiments. **a and b** The data of the Figure 2 obtained with metaphase-synchronized cells are shown for comparison.
