# Supplementary material for: Mitotic arrest affects clustering of tumor cells
Source: Cell Div. 2021 Jan 29;16:2. doi: 10.1186/s13008-021-00070-z (PMC7847029; doi:10.1186/s13008-021-00070-z)
Supplement: Supplementary file 3 — Additional file 3: Figure S3. MCF-7 cell clustering occurs independently of microtubules cytoskeleton disruption. a Immunostaining of -tubulin on control untreated MCF-7 cells (Control) and cells incubated with 10 µM nocodazole for 2 h. For both conditions, each row corresponds to two different fields of view. Inserts show the higher magnification of the region outlined with dotted lines in the corresponding image. *mitotic cells in control and nocodazole conditions. Scale bar: 10 µm. b Schematic representation of the experiment. To test the impact of microtubule depolymerization on aggregation dynamics, MCF-7 cells were pre-treated with 10 µM nocodazole for 1 h, then they were seeded in 96-well low-attachment plates in presence of 10 µM nocodazole, and monitored by video-microscopy for 5 h (clustering assay). c Percentage of compaction calculated from the normalized area at each time points (see “Methods” section) for control (untreated) cells and cells incubated with nocodazole, as described in b. For each time point, data correspond to the mean ± SD of 32 aggregates/condition from 3 independent experiments. [file 13008_2021_70_MOESM3_ESM.pdf]

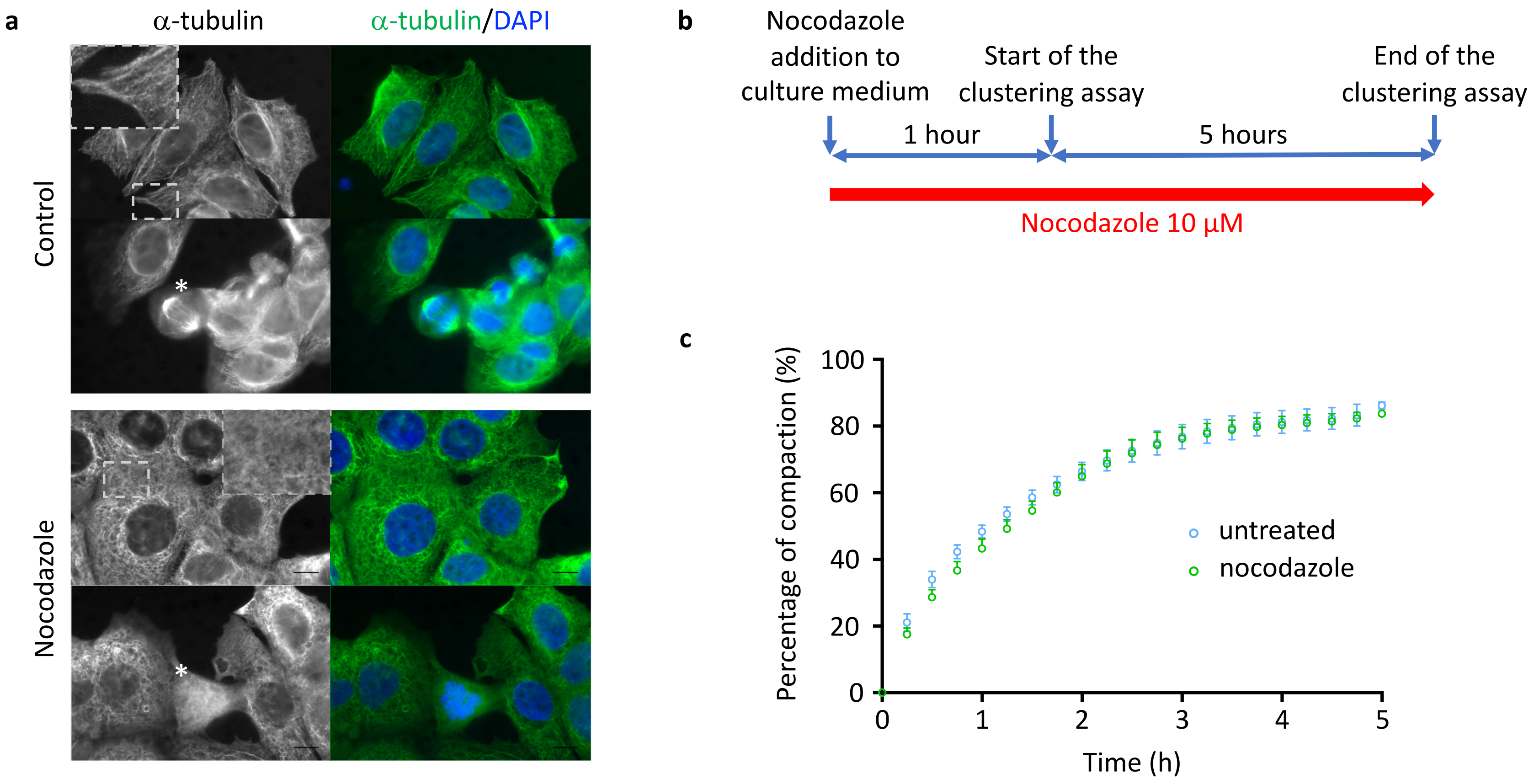

Supplementary Figure S3: MCF-7 cell clustering occurs independently of microtubules cytoskeleton disruption

a Immunostaining of α-tubulin in untreated MCF-7 cells (Control) and cells incubated with 10 µM nocodazole for 2 hours. For both conditions, each row corresponds to two different fields of view. Inserts show the higher magnification of the region outlined with dotted lines in the corresponding image. *: mitotic cells in control and nocodazole conditions. Scale bar: 10 µm. **b** Schematic representation of the experiment. To test the impact of microtubule depolymerization on aggregation dynamics, MCF-7 cells were pre-treated with 10 µM nocodazole for 1 h, then they were seeded in 96-well low-attachment plates in the presence of 10µM nocodazole, and monitored by videomicroscopy for 5 hours (clustering assay). **c** Percentage of compaction calculated from the normalized area at each time point (see Methods section) for control (untreated) cells and cells incubated with nocodazole, as described in b. For each time point, data correspond to the mean ± SD of 32 aggregates/condition from 3 independent experiments.
