# Supplementary material for: Mitotic arrest affects clustering of tumor cells
Source: Cell Div. 2021 Jan 29;16:2. doi: 10.1186/s13008-021-00070-z (PMC7847029; doi:10.1186/s13008-021-00070-z)
Supplement: Supplementary file 4 — Additional file 4: Figure S4. Aggregation ability of cells accumulated in mitosis independently of microtubule cytoskeleton disruption is also altered. a Flow cytometry analysis of control (untreated) cells, cells incubated with 6 µM RO3306 (RO3306) for 20 h, or cells at the indicated time points after RO3306 removal from the culture medium. The upper panels show the histograms of the propidium iodide fluorescence intensity (DNA content) and the lower panels show the dot plots of DNA content versus intensity for the detection of the mitotic marker 3.12.I.22 (see “Methods” section). The percentage of mitotic cells (shown in green) is indicated for each condition. b Schematic representation of the experimental design for the aggregation assay. c The percentage of compaction was calculated at each time point of the clustering assay in control cells and in cells collected by shake-off at 2 h after RO3306 removal. Data correspond to the mean ± SD of 32 aggregates in control and 35 aggregates in treated cells from 3 independent experiments. [file 13008_2021_70_MOESM4_ESM.pdf]

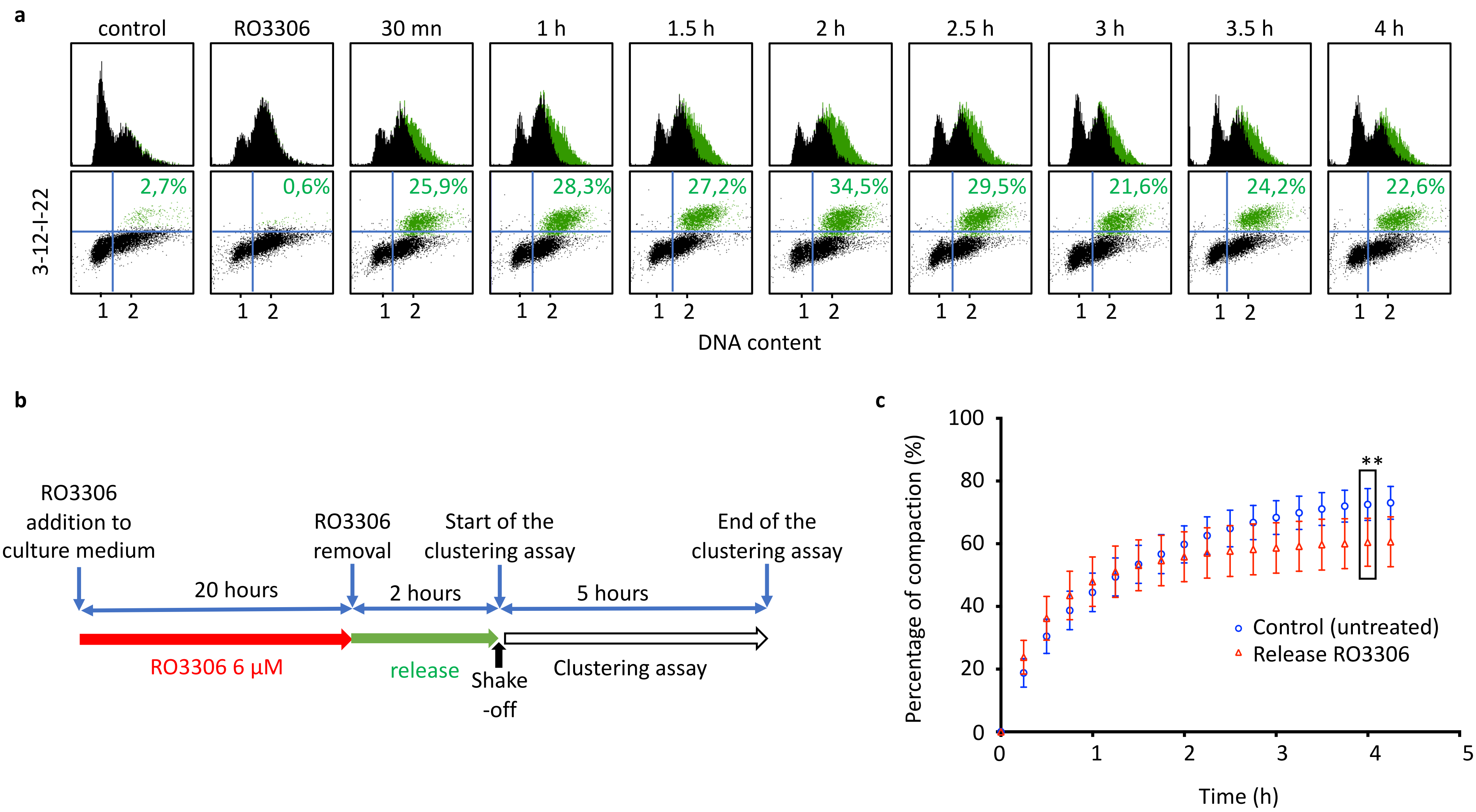

Supplementary Figure S4: Aggregation ability of cells accumulated in mitosis independently of microtubule cytoskeleton disruption is also altered

a Flow cytometry analysis of control (untreated) cells, cells incubated with 6 µM RO3306 (RO3306) for 20h or cells at the indicated time points after RO3306 removal from the culture medium. The upper panels show the histograms of the propidium iodide fluorescence intensity (DNA content) and the lower panels show the dot plots of DNA content versus intensity for the detection of the mitotic marker 3.12.I.22 (see methods section). The percentage of mitotics cells (shown in green) is indicated for each condition. **b** Schematic representation of the experimental design for the aggregation assay. **c** The percentage of compaction was calculated at each time point of the clustering assay for control cells and cells collected by shake-off at 2 hours after RO3306 removal. Data correspond to the mean ± SD of 32 aggregates in control and 35 aggregates in treated cells from 3 independent experiments.
