# Supplementary material for: Mitotic arrest affects clustering of tumor cells
Source: Cell Div. 2021 Jan 29;16:2. doi: 10.1186/s13008-021-00070-z (PMC7847029; doi:10.1186/s13008-021-00070-z)
Supplement: Supplementary file 5 — Additonal file 5: Figure S5. Microdevice to study clustering at the single-cell scale. a Mask used for the fabrication of the silicon wafer. b One array of 9 PDMS micro-wells (outer diameter: 650 µm, inner diameter: 450 µm, and height: 200 µm) that are (c) glued on the bottom of the compartments of CELLviewTM cell culture dishes for monitoring by time-lapse video-microscopy. [file 13008_2021_70_MOESM5_ESM.pdf]

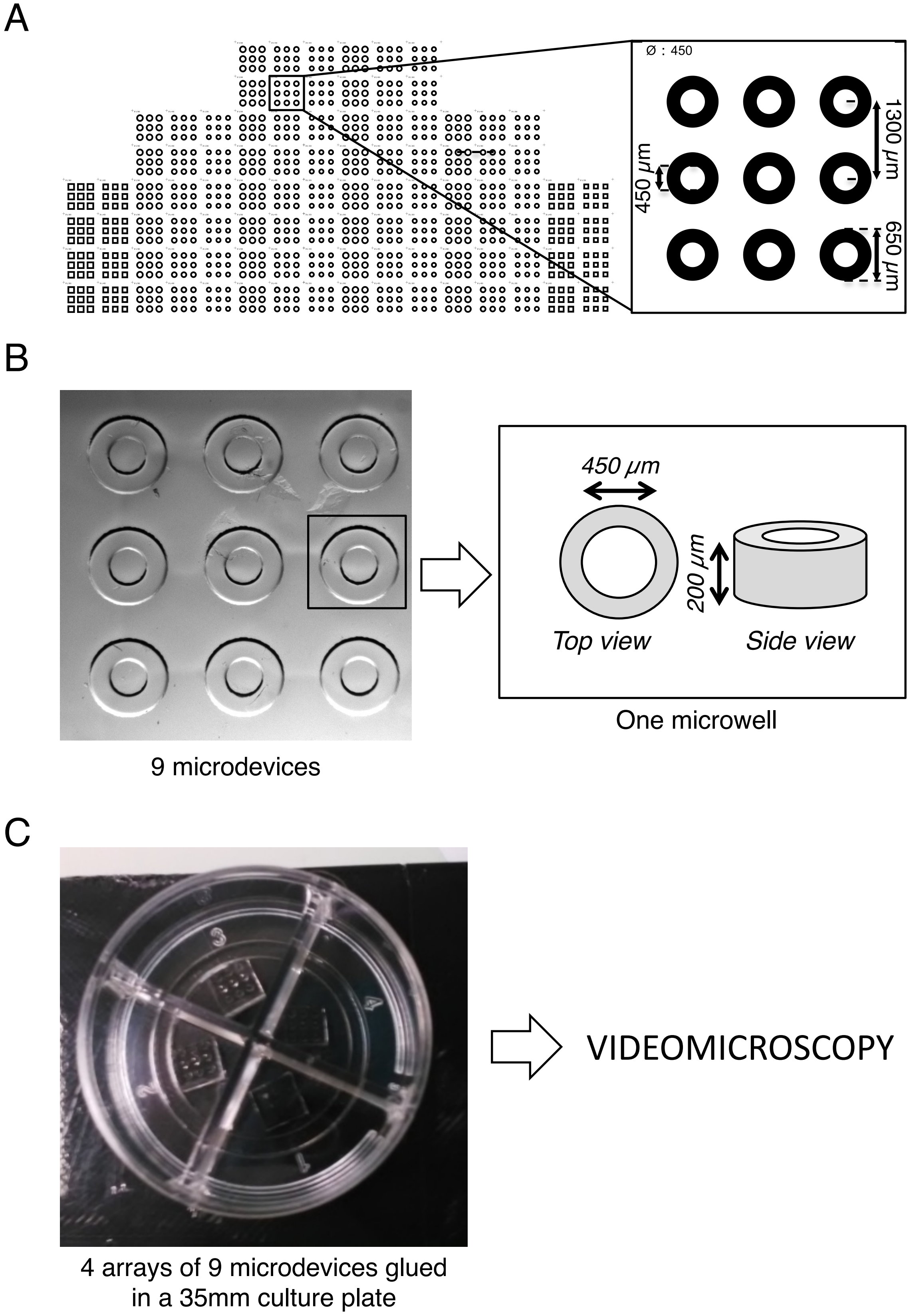

Supplementary Figure S5. Microdevice to study clustering at the single-cell scale

a Mask used for the fabrication of the silicon wafer. **b** One array of 9 PDMS micro-wells (outer diameter: 650µm, inner diameter: 450µm, and height: 200µm) that are (**c**) glued on the bottom of the compartments of CELLview^{TM} cell culture dishes for monitoring by time-lapse video-microscopy.
