# Supplementary material for: Mitotic arrest affects clustering of tumor cells
Source: Cell Div. 2021 Jan 29;16:2. doi: 10.1186/s13008-021-00070-z (PMC7847029; doi:10.1186/s13008-021-00070-z)
Supplement: Supplementary file 10 — Additional file 10: Figure S6. Characterization of clusters in control and experimental conditions. a, b Graphs showing the aspect ratio (a) and circularity (b) analysis results for the larger clusters formed in micro-wells in MCF-7 cells incubated or not (untreated, UNT) with nocodazole and MG132 (i.e., metaphase-synchronized/blocked, Met-sync), or with MG132 (MG) or nocodazole (Noco) alone after 3 h of clustering. Each dot corresponds to the values in one micro-well from 5 independent experiments and bars correspond to the mean ± SD. c, d Determination of the average (µ) (c) and standard deviation (σ) (d) aspect ratio in single MCF-7 cells incubated or not (untreated, UNT) with nocodazole and MG132 (i.e., metaphase-synchronized/blocked, Met-sync), or with MG132 (MG) or nocodazole (Noco) alone after 1 h of aggregation. Each dot corresponds to one cell and the bars correspond to the mean ± SD. Data are from 5 independent experiments with 5-6 cells analyzed per experiment. *P < 0.05; **P, < 0.01; ****P < 0.0001 (Mann–Whitney non-parametric test). [file 13008_2021_70_MOESM10_ESM.pdf]

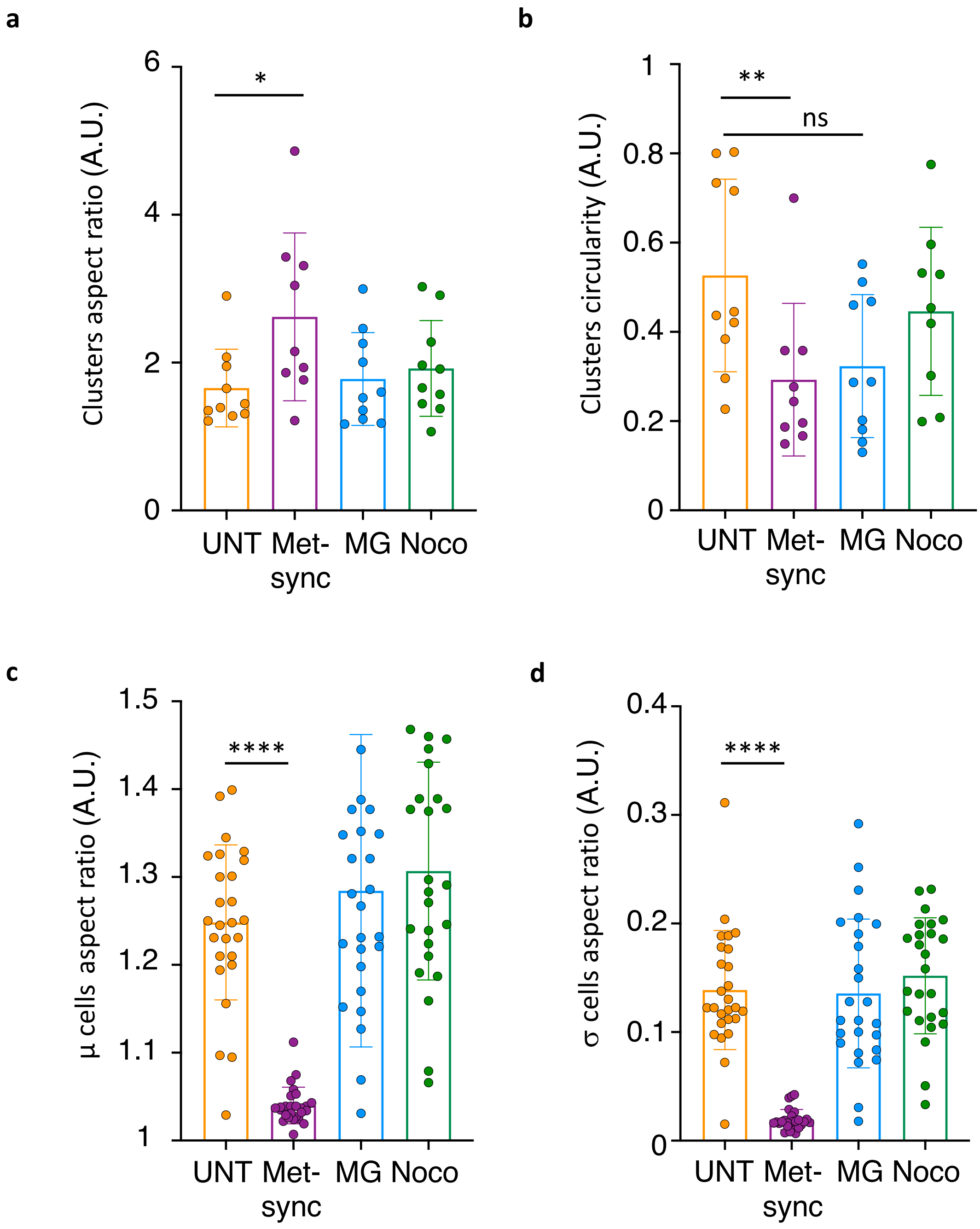

Supplementary Figure S6. Characterization of clusters in control and experimental conditions.

a, b Graphs showing the aspect ratio (**a**) and circularity (**b**) analysis results for the larger clusters formed in micro-wells in MCF-7 cells incubated or not (untreated, UNT) with nocodazole and MG132 (i.e., metaphase-synchronized/blocked, Met-sync), or with MG132 (MG) or nocodazole (Noco) alone after 3 hours of clustering. Each dot corresponds to the values in one micro-well from 5 independent experiments and bars correspond to the mean ± SD. **c,d** Determination of the average (μ) (**c**) and standard deviation (σ) (**d**) aspect ratio in single MCF-7 cells incubated or not (untreated, UNT) with nocodazole and MG132 (i.e., metaphase-synchronized/blocked, Met-sync), or with MG132 (MG) or nocodazole (Noco) alone after 1 hour of aggregation. Each dot corresponds to one cell and the bars correspond to the mean ± SD. Data are from 5 independent experiments with 5-6 cells analyzed per experiment. *, $P<0.05$; ** P, <0.01; ****, $P <0.0001$ (Mann Whitney non-parametric test).
